# Reversal of Toric Intraocular Lens-corrected Corneal Astigmatism after Kahook Dual Blade Goniotomy

**DOI:** 10.5005/jp-journals-10078-1243

**Published:** 2019

**Authors:** Matthew T Hirabayashi, Lindsey M McDaniel, Jella A An

**Affiliations:** 1 Department of Ophthalmology, University of Missouri School of Medicine, Columbia, Missouri, USA; 2,3 Department of Ophthalmology, University of Missouri School of Medicine, Columbia, Missouri, USA; Mason Eye Institute, University of Missouri, Columbia, Missouri, USA

**Keywords:** Astigmatism, Kahook dual blade, Phaco, Toric

## Abstract

A 79-year-old man underwent phacoemulsification (phaco) with TORIC intraocular lens (IOL) insertion combined with Kahook dual blade (KDB) goniotomy of the right eye several months after a stand-alone phaco in the fellow eye. He had significant against-the-rule astigmatism in both eyes (2.41D @ 10° right, 2.40D @ 160° left) preoperatively. Postoperatively, nearly all corneal astigmatism disappeared in the right eye (0.60D @ 37°), while it remained the same in the left eye (2.00D @ 167°). Ophthalmologists should be aware that KDB may have an unreported effect of altering corneal astigmatism, which should be considered when inserting TORIC IOL.

**How to cite this article:**

Hirabayashi MT, McDaniel LM, *et al.* Reversal of Toric Intraocular Lens-corrected Corneal Astigmatism after Kahook Dual Blade Goniotomy. J Curr Glaucoma Pract 2019;13(1):42–44.

## INTRODUCTION

Kahook dual blade (KDB) goniotomy (New World Medical, Rancho Cucamonga, CA, 2015) has become one of the most popular angle surgeries to date. Its dual blade design allows complete removal of the diseased trabecular meshwork (TM) via an ab-interno approach to decrease aqueous outflow resistance.^1^ The effect of KDB on post-op corneal astigmatism is yet to be studied.

## CASE DESCRIPTION

**Fig. 1:**
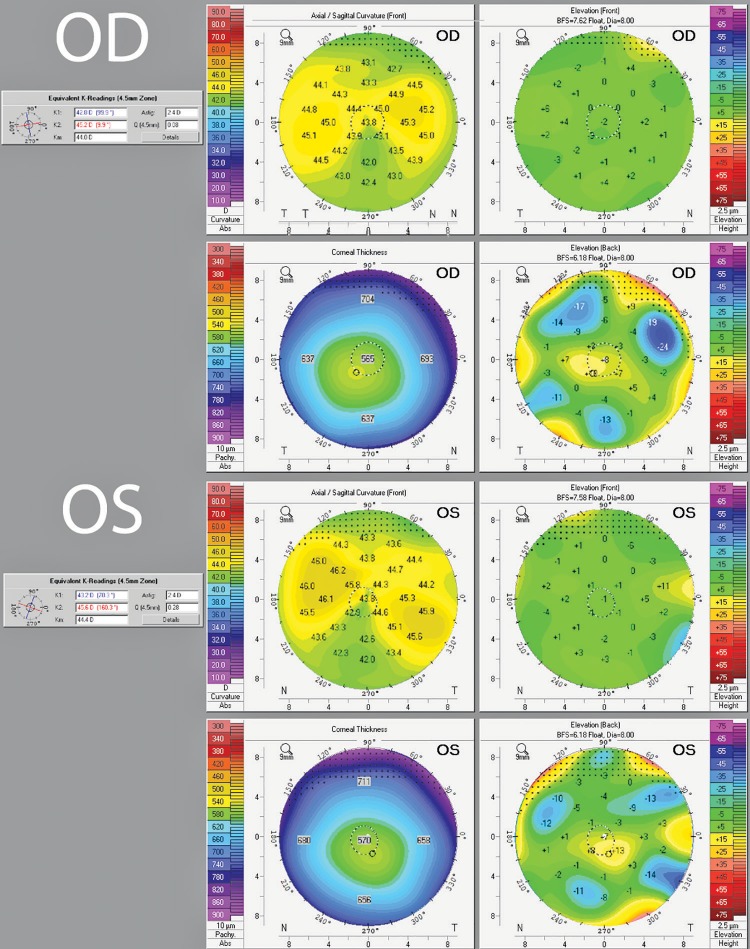
Preoperative topography showed significant against the rule astigmatism in both eyes of 2.4D @ 9.9° OD and 2.4D @ 160.3° OS

Here, a 79-year-old male presented for phacoemulsification (phaco) combined with KDB goniotomy of the entire nasal angle of the right eye (OD) and phaco alone of the left eye (OS). He had a history of advanced primary open-angle glaucoma (POAG) in both eyes. He was noted to have significant against-the-rule corneal astigmatism in both eyes (2.41D @ 10° OD, 2.40D @ 160° OS) preoperatively (Fig. 1). His pre-op autorefraction showed −0.24 + 1.75 × 006 OD and −0.25 + 1.25 × 160 OS and pre-op IOP was 23 OU. A ZCT 400 18 D toric lens (Bausch and Lomb, Bridgewater, New Jersey) was placed at axis 165° in OS (target −0.33), followed months later by a ZCT 400 18.5D toric lens at axis 10° in OD (target −0.48), both without complication. Both incisions were at 180° and 0° for each eye.

On postoperative day 7 after OD surgery, he presented to the clinic for blurry vision. IOP was 13 mm Hg in OD and 17 mm Hg in OS. Uncorrected visual acuity in Snellen was 20/70 OD (20/40 pinhole) and 20/40 OS. Exam and OCT of the macula were unremarkable. Dilated exam revealed that both lenses had rotated—the right lens from 10° to 165° and the left lens from 165° to 180°. Interestingly, repeat topography showed that the right eye astigmatism had also changed from 2.41D @ 10° to 1.5 @ 80°, while the astigmatism of the left eye remained the same as preoperative measurements. One month later, the right lens had shifted slightly further to 170° and the left lens moved to 20°. Manifest refraction showed −0.75 + 0.25 × 045 (20/20) OD and −0.25 + 1.25 × 054 (20/20) OS. His IOP remained stable and the patient was happy.

**Fig. 2:**
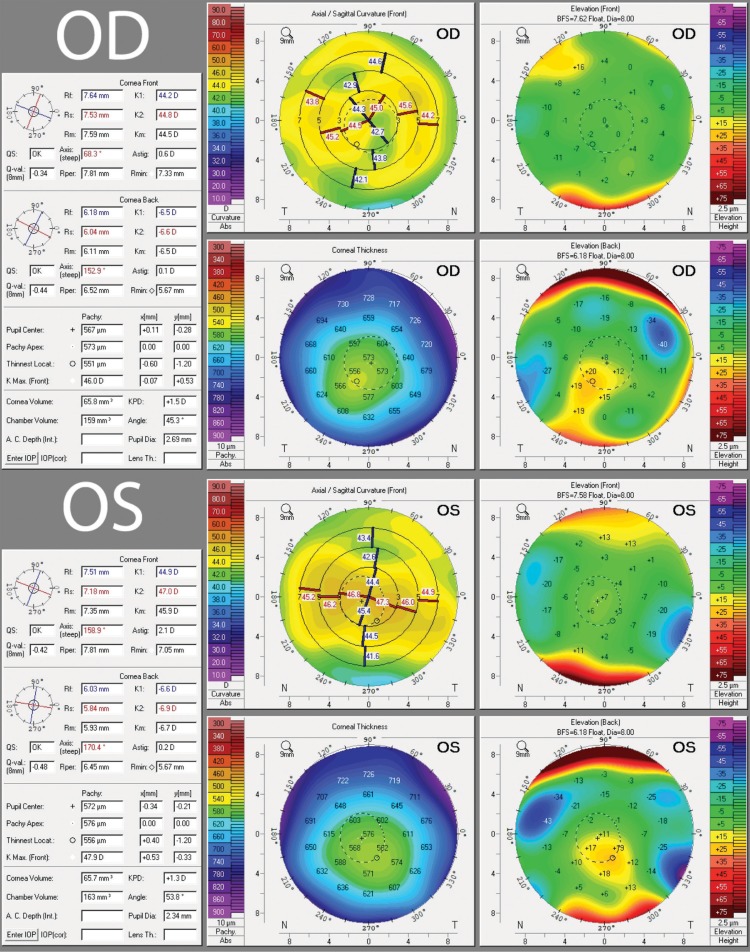
Postoperative topography 4 months after the second eye showed resolution of OD astigmatism to 0.60D @ 37° while OS remained unchained at 2.1D @ 167°. Axis measurements were derived from Holladay report for accuracy (not pictured here)

After 4 months of observation, repeat topography revealed that his OD astigmatism had further shifted from the preoperative 2.41D @ 10° to postoperative 0.6D @ 37°, while his left eye changed only slightly from 2.4D @ 160 to 2.06D @ 167° (Fig. 2), without any topographical or clinical sign of corneal dystrophy. Fortunately for this patient, the shift in his corneal astigmatism in OD was offset by the rotation of the toric lens. The patient still had 1.25D of residual astigmatism at 54° OS but was satisfied with vision corrected by glasses in that eye.

## DISCUSSION

Due to the anatomical relationship between the TM, peripheral iris, anterior chamber angle, and posterior cornea, patients who undergo near-total removal of their sectoral TM may experience changes in any existing posterior corneal astigmatism in that axis. To our knowledge, no studies have evaluated the effect of KDB goniotomy on corneal astigmatism, but a recent report showed phaco-KDB was no more likely to result in refractive surprise than phaco alone.^2^

This case is unique, as the patient had a fellow eye to serve as a control, which did not show changes in corneal astigmatism after phaco alone. This patient's postoperative topography showed near-complete resolution of his preoperative against-the-rule astigmatism in OD where he had nasal TM removed with KDB goniotomy. Remarkably, rotation of his TORIC IOL essentially negated its effect, leading to near-plano uncorrected visual acuity of 20/20 OD. If KDB does truly affect corneal astigmatism, then clinicians must consider when choosing to implant TORIC IOLs. Further studies to investigate the exact patterns and amount of astigmatism change will help determine the KDB-related surgeon factor to predict better outcomes of TORIC IOLs.

## References

[1] Salina L,, Chaudhary A, (2018;). Goniotomy Using the Kahook Dual Blade in Severe and Refractory Glaucoma: Six Month Outcomes.. J Glaucoma.

[2] Young C,, Sieck E, (2019;). Refractive Outcomes Among Glaucoma Patients Undergoing Phacoemulsification Cataract Extraction with and Without Kahook Dual Blade Goniotomy.. Poster presented at the: American Glaucoma Society 29th Annual Meeting;.

